# Prevalence and Associated Factors of Depression, Anxiety, and Stress in University Students in Paraguay during the COVID-19 Pandemic

**DOI:** 10.3390/ijerph191912930

**Published:** 2022-10-09

**Authors:** Telmo Raul Aveiro-Róbalo, Luciana Daniela Garlisi-Torales, Marisella Chumán-Sánchez, César J. Pereira-Victorio, Mariana Huaman-Garcia, Virgilio E. Failoc-Rojas, Mario J. Valladares-Garrido

**Affiliations:** 1 Facultad de Medicina, Universidad del Pacífico, Asunción 2300, Paraguay; 2 Dirección de Investigación 2300, Universidad Maria Auxiliadora, Asunción 2040, Paraguay; 3 Sociedad Científica de Estudiantes de Medicina Veritas (SCIEMVE), Escuela de Medicina, Universidad San Martín de Porres, Chiclayo 14012, Peru; 4 School of Medicine, Universidad Continental, Lima 12000, Peru; 5 Escuela de Medicina, Universidad Cesar Vallejo, Piura 20001, Peru; 6 Research Unit for Generation and Synthesis Evidence in Health, Universidad San Ignacio de Loyola, Lima 15024, Peru; 7 South American Center for Education and Research in Public Health, Universidad Norbert Wiener, Lima 15046, Peru; 8 Epidemiology Office, Hospital Regional Lambayeque, Chiclayo 14012, Peru

**Keywords:** depression, anxiety, stress, COVID-19, Paraguay

## Abstract

We aimed to determine the prevalence of depression, anxiety, and stress in university students in Paraguay during the COVID-19 pandemic. A cross-sectional study was conducted among 293 students from four universities in Paraguay in 2021. The DASS-21 mental health scale was used to measure the outcomes (depression, anxiety, and stress) and evaluate their association with socio-educational variables. A total of 77.1% of the participants were women and 136 (46.4%) were between 21 and 25 years old. The prevalence of depression, anxiety, and stress was 74.7%, 87.4%, and 57%, respectively. We found that being a woman and studying at a public university was positively associated with depression, anxiety, and stress. Receiving COVID-19 training increases the prevalence of mental health problems. In conclusion, high levels of anxiety, depression, and stress were found in university students during the COVID-19 pandemic. Being a woman, studying at a public university, and receiving training on COVID-19 were factors associated with a higher prevalence of presenting all the mental health problems evaluated. Furthermore, students aged 31 and over had a higher prevalence of depression and stress.

## 1. Introduction

The coronavirus pandemic has directly influenced the mental health of different populations [1,2,3,4,5,6,7,8,9]. In recent years, there has been a growing interest in researching stress, anxiety, and depression in different contexts, such as at work, at universities, and in families [10,11,12], which has led to decision-making and specific strategies to improve these problems. Along these lines, stressful situations such as the pandemic can precipitate the manifestation of these pathologies [13].

It is known that health sciences students have a higher prevalence of mental health disorders compared to other careers, due to academic demands, emotional exhaustion, and social isolation [14,15,16]. Therefore, the COVID-19 pandemic might have negatively affected mental health, and thus, affected performance, professional development, and interpersonal relationships [17,18]. There is an association between university students’ stress levels and emotional disorders due to the absence of programs that mitigate emotional problems, such as depression and anxiety [14]. Social isolation causes stress that can be directly associated with resistance to glucocorticoids, proinflammatory expression, and oxidative stress, [19], which can also occur in cases of depression and in perceptions of loneliness [20].

The world prevalence of depression or depressive symptoms among medical students is 27.2% [12], while the prevalence of anxiety is 28% [15] and stress is 23% [21]. However, there is still insufficient evidence in Latin America on the factors that explain the development of mental health problems in university students as a consequence of the COVID-19 pandemic [22]. In Paraguay, there has been no relevant information to date that addresses this problem in young people; more evidence is needed in this region due to the instability of health systems and social inequities.

The objective of this research is to determine the prevalence of depression, anxiety, and stress in university students (studying Architecture, Health Sciences, Administration, Agriculture, Social Sciences, Engineering, Technology, and Physics) in Paraguay during the COVID-19 pandemic, 2021.

## 2. Materials and Methods

### 2.1. Study Design

We conducted an analytical cross-sectional study, with the aim of determining the prevalence and factors associated with depression, anxiety, and stress in university students from Paraguay during the period from June to August of the year 2021.

Data collection was conducted during the period from June 9 to 25 August 2021. Regarding the pandemic situation during this period, the number of new confirmed cases per day and number of registered deaths from COVID-19 were quite high. In the month of June, more than 1000 cases per day were confirmed, and from July 15, the number of new confirmed cases per day decreased to approximately 500 cases per day [23]. Likewise, the number of deaths from COVID-19 registered per day in the month of June exceeded 100 and a high mortality rate was recorded throughout the month of June [24,25].

Quarantine restrictions were softened during the first half of June, with the entire country entering phase 3 of the smart quarantine. At the end of July, the country entered phase 4 of the smart quarantine. During phase 3, people could move freely within the national territory, with a curfew from 23:00 to 05:00. In phase 4, the rules regarding enclosed places and the number of people who could stay in different places were made to be more flexible [26].

Throughout the data collection period, students had classes through digital platforms and some face-to-face field practices in small groups with all the sanitary measures imposed by the country’s health ministry [26].

### 2.2. Population and Sample

The population was made up of university students from four universities in Paraguay. The sample was 293 students of health sciences and others university careers. All those who agreed to participate in the study and who answered the questions of interest correctly were included. Minors under 18 years old were excluded. A non-probabilistic convenience sampling was applied. A representative sample of Paraguayan university students was calculated with Epi Info 7.0 program (Centers for Disease Control and Prevention, Atlanta, GA, USA) using the formula for an infinite population, with an estimated proportion of 84.52% [27], a confidence level of 95%, and a precision of 5%, which gave us a minimum respondent size of 202. To this, we added 20% for refusals and 20% for incomplete surveys. Therefore, the estimated sample was 283. However, we were able to capture a larger number of students (*n* = 293).

### 2.3. Variables

The dependent variable was depression, anxiety, and stress. Depression was defined as a score greater than 4 points obtained from the sum of the DASS-21 test responses. Anxiety was defined as a score of 5 points or more in the DASS-21 test answers. Stress was operationally defined as a score greater than 7 points obtained by the DASS-21 questionnaire.

The independent variables were socio-academic variables, such as age, sex, marital status, university degree, and academic year, in addition to questions related to the period of social distancing experienced during the pandemic, such as the number of people with whom they lived in their accommodation and the number of COVID-19 training sessions they received during that period.

### 2.4. Instrument

The DASS-21 instrument was used. It was made up of 21 items and had 7 items for each subscale to measure the three mental health outcomes (depression, anxiety, and stress). It had adequate psychometric properties for use in Chilean university students (Cronbach’s alpha 0.85, 0.73, and 0.83 for depression, anxiety, and stress, respectively). [28] The instrument defines stress as an emotional state that varies according to individuals’ assessment of situations experienced as threatening, harmful, or challenging. The DASS-21 assesses emotional demand and coping strategies, with symptoms and emotions serving as the main organizing concepts of the experience of stress.

### 2.5. Procedures

After obtaining the corresponding permits, we created a survey on the Google Forms platform to generate a virtual data collection instrument. This instrument was shared by institutional social networks of the participating universities. The data obtained were subsequently exported in a data sheet using the Microsoft Excel program for further analysis.

### 2.6. Data Analysis

In the descriptive analysis, absolute and relative frequencies were estimated for the categorical variables. In the bivariate analysis, the chi-square test was used, after evaluating the assumption of expected frequencies. Simple and multiple regression models were built to evaluate the factors associated with depression, anxiety, and stress. In the multiple regression model, the covariates that were associated in the simple model were entered (*p* < 0.05). Prevalence ratios (PR) and 95% confidence intervals (95% CI) were estimated by using generalized linear models, Poisson distribution family, log link function, robust variance, and year of studies (schooling) as clusters. Data were analyzed in Stata^®^ 17 software (StataCorp LP, College Station, TX, USA).

### 2.7. Ethical Aspects

The research protocol was approved by the ethics committee of the Universidad del Pacífico, Paraguay. The survey was conducted anonymously, and informed consent was requested from each respondent.

## 3. Results

Of 293 university students surveyed, 77.1% were women, 46.4% were between 21 and 25 years old, 52.9% were from a private university, and 58.7% were studying a health career. A total of 46.1% had received at least one training session or talk about COVID and 21.8% lived with at least five people in their accommodation. Almost half (49.8%) presented extremely severe anxiety and 30.4% presented moderate depression. A total of 17.8% presented severe stress (Table 1).

Age was associated with depression (*p* = 0.002) and stress (*p* = 0.030). Higher frequencies of depression, anxiety, and stress were found in females compared to males, showing significant differences (*p* < 0.001). Single students had a higher frequency of depression compared to non-single students (76.7% vs. 55.6%; *p* = 0.016). Coming from a public university was associated with a higher frequency of depression (*p* < 0.001), anxiety (*p* = 0.003), and stress (*p* = 0.004), as shown in Table 2.

In the multiple regression model, we found that the factors associated with a lower prevalence of depression were as follows: being between 26 and 30 years old (PR = 0.82; CI95%:0.65–0.76); being 31 years or older (PR = 0.70; 95% CI: 0.65–0.76); and being male (PR = 0.67; 95% CI: 0.4–0.95). In contrast, studying at a public university and having received a minimum of five training sessions on COVID increase the prevalence of depression by 31% (PR = 1.31; 95% CI: 1.09–1.58) and 56% (PR = 1.56; 95% CI: 1.20–2.02), respectively. Additionally, we found that men have a 33% lower prevalence of anxiety (PR = 0.67; 95% CI: 0.58–0.76). The factors positively associated with anxiety were the type of public university (PR = 1.16; 95% CI: 1.09–1.24), and having received a minimum of three training sessions on COVID-19 (PR = 1.06; 95% CI: 0.99–1.13). Moreover, being 31 years old or older (PR = 1.29; 95% CI: 1.15–1.43), studying at a public university (PR = 1.35; 95% CI: 1.08–1.68), and receiving a minimum of five training sessions on COVID-19 (PR = 1.74; 95% CI: 1.27–2.40) were associated with a higher prevalence in stress. In contrast, a factor negatively associated with stress was being of the male gender (PR = 0.47; 95% CI: 0.37–0.60) Table 3. 

## 4. Discussion

### 4.1. Prevalence of Mental Health Disorders

In our study, it was found that almost eight out of every ten students presented anxiety (87.4%). This is similar to the results reported by an American study, in which the prevalence was more than 60% [29]. However, it differs from what was found in previous studies carried out in Brazil and Iran, in which the prevalence of anxiety was 52.5% [30] and 38.1% [31], respectively. This finding could be explained by the fact that most of our studied population is health sciences students, who are more likely to have anxious symptoms due to the constant stress they face, causing negative repercussions on their psychosocial well-being [19,32]. Likewise, the COVID-19 pandemic has probably had an impact on the population evaluated, as corroborated by the literature, which states that the pandemic increased previous anxious symptoms, especially somatic ones [31]. This finding could explain why students with higher levels of anxiety are more likely to make excessive purchases of hygiene utensils and visit hospitals for ailments without much clinical relevance [33], while those with less anxiety may ignore biosafety measures with a “lack of fear” of becoming infected [34]. Despite the predisposing factors for the development of anxiety caused by the pandemic, systematic reviews reveal a low prevalence of anxiety in students before (27.22%) [35] and during (28%) [15] the COVID-19 outbreak.

Notably, the prevalence of extreme severe anxiety was 49.8%, indicating almost half of the participants. This result could be explained by the higher frequency of stressful events that occurred during the pandemic. However, this finding could be the result of the low specificity of the instrument, which would result in a misclassification of the severity of anxiety.

In addition, seven out of every ten students had depression (74.7%). This finding is similar to that reported by a study conducted in the United States, in which the frequency of depression was 80.6% [36]. However, it differs from what was found by Simegn W. et al., [37] Nakhostin-Ansari A. et al., [31] and Gan G.G. et al., [38] whose studies revealed a depression prevalence of 46.3%, 27.6%, and 11%, respectively. Systematic reviews carried out before the pandemic reveal a prevalence of 51.5% [39] and 27.2% [35].

This finding could be explained by the fact that, unlike other countries, the Latin American student population has more marked problems on economic, political and social levels [40]. In addition, the arrival of COVID-19 brought with it preventive measures, such as mandatory social distancing, which completely transformed the routine to which the population was accustomed, reducing their physical contact with social groups and in turn triggering changes at the social level, emotionally, and even the development of depressive disorders [41]. Although confinement allowed family members to have closer relationships with each other [42], changes in education and work generated internal conflicts due to the need to share electronic devices and spaces within the home to carry out their activities, which impacted the emotional states of each of the members [43].

Additionally, almost five out of every ten students presented stress (57.0%). This is similar to what was reported by a study in Brazil (57.5%) [30] and a study conducted before the pandemic (53%) [44]. However, it differs from what was found in two Peruvian studies, in which the general population was evaluated at the beginning of the pandemic and the stress prevalence was found to be 15% [45] and 13% [22]. This finding could be explained by the overload of tasks and the process of adaptation to the new virtual evaluation system that university students had to face during the first months of the pandemic as a consequence of the closures of universities, which was not only reflected in their stress levels, but also in their symptoms, such as headaches, migraine, and chronic fatigue [46].

### 4.2. Factors Associated with Depression

We found that students 31 years and older had a 30% lower depression prevalence. This differs from what was found by Huarcaya-Victoria J. et al., [47] whose study revealed that students older than 25 years and those who are in the 18–25 year range have more depressive symptoms. However, there are studies that do not report statistical significance between age and depressive symptoms [48].

Males had a 37% lower prevalence in depression. This is similar to the results reported by a multicenter study conducted in Peru [47]. However, other authors have reported no significant difference in terms of gender and depression [49]. This finding could be explained by the fact that mental health disorders predominate in women due to the responsibilities they acquire from an early age, in addition to risk factors such as exposure to sexual abuse and domestic violence [50,51]. In addition, they tend to present hyperactivity and cognitive disorders to a greater extent [52]. Meanwhile, the male population usually presents behaviors such as irritability, impulsiveness, and certain addictions, which can mask depressive symptoms [53].

Studying at a public university is a factor that represents a 31% higher prevalence of depression. This is similar to the results reported by Mehareen J. et al. [54]. This finding could be explained by clear differences between private and public universities, the latter being less equipped both in infrastructure and in technological tools, which could have meant a delay in adapting to virtual education during the first months of the pandemic, therefore increasing the uncertainty of university students and translating into depressive symptoms [54,55].

Students who reported having received five training sessions on COVID-19 had a 56% higher prevalence of depression. This is similar to what was found in a study by Galić M. et al. [56]. This can be explained because the information received through the training can be translated into different behaviors: if the information received is positive, it will promote rational coping behaviors. However, if it is negative, it could increase the level of risk perception, resulting in the appearance of irrational fear and probable depressive symptoms [57].

### 4.3. Factors Associated with Anxiety

Male students had a lower prevalence of anxiety. This is similar to what was reported by Sheshtawy HA et al. [58]. However, it contradicts what was found by Yuan L.L. et al. [59] and by Simegn W. et al. [37]. This finding could be explained by the fact that women tend to report appetite and sleep disorders, tiredness, anxiety, and hypochondria more frequently [51]. In addition, the difference in the types of personalities between each gender tends to be noticeable. Men tend to have more objectivity, focus on problems, and are able to find quick solutions, which is why they could be less susceptible to suffering from certain mental disorders, such as anxiety [60].

Attending a public university was positively associated with anxiety. This is similar to the results reported by Hossain MA et al. [61]; however, in a study conducted before the pandemic, students from a private university had more anxiety [62]. This could be explained by the psychological pressure caused by factors, such as academic delays and instability in family incomes, which tends to be seen more frequently in the homes of students attending public universities [63].

Students who reported receiving five COVID-19 training sessions had a 12% higher prevalence of anxiety. However, this contradicts what was found in a study by Doraiswamy S. et al. [64] and Reddy P. et al. [65], in which having a good level of knowledge about COVID-19 was associated with low levels of anxiety.

### 4.4. Factors Associated with Stress

Being 31 years old or older was a factor that represented a higher prevalence of stress in the participating students. However, this contrasts with what was found by Sandoval KD et al. [22], whose study revealed that younger students had a higher prevalence of stress than older ones. This finding could be explained by the fact that older university students are usually in the last years of their medical degree, and therefore, they take more rigorous academic subjects than in the first years of their career. In addition, the increased responsibilities that adult life brings could increase the students’ stress level. However, those who are in the first years of their degree are also exposed to stressful factors, as part of the adaptation process when starting university [22]. It is important to highlight that management strategies to cope with stress usually influence the appearance of stressful symptoms [66], so that, regardless of age, this factor could have affected the results.

Males had a lower prevalence of stress. This is similar to the results reported by other studies [67,68]. This finding could be explained because various studies have shown that women expressed greater stress levels than male students before the COVID-19 pandemic [69,70] and even during the Influenza AH1N1 pandemic [71]. This could also be explained because women tend to take problems and negative events personally, unlike men, who take more immediate action in the face of difficulties [72].

Coming from a public university is positively associated with having stress. This is similar to the results reported by Hossain MA et al. [61]. However, it contradicts what was found before the pandemic [62] in a study that showed 17.4% of the students from public universities were “extremely stressed”. These findings may be explained by the differences in evaluation methodology between public and private universities; likewise, students from public universities could be more exposed to student pressure to meet the expectations of their parents regarding their academic performance [62].

Students who reported receiving five COVID-19 training sessions had a 74% higher prevalence of stress. However, this is in contrast to what was found in a study carried out in Mexico [73]. This should be explained by the fact that receiving too much information can often lead to the development of stress symptoms, or the worsening previous mental health problems [74].

### 4.5. Implications of Findings in Mental Health

The results obtained in this research indicate the impact that the COVID-19 pandemic has had on the mental health of the university population in Paraguay. The presence of a high prevalence of neurotic disorders in this population has not been recorded before and generates concern and challenges for Paraguayan universities [29]. Therefore, it is recommended that university authorities implement educational actions that promote effective coping techniques and enhance mental well-being [30]. In addition, clinical and community psychosocial interventions [41] are needed to enable students to develop appropriate strategies that provide comprehensive psychosocial care [29].

### 4.6. Limitations and Strengths

In this research, we found some limitations. First, there is a selection bias, because a non-probabilistic sampling was used and our research has included only four universities from Paraguay, so the results cannot be extrapolated to the entire study population. Second, due to the cross-sectional design, it is not possible to attribute causality between the variables of interest evaluated. Third, there is a probable measurement bias, since it was not possible to explore other variables that explain the mental health outcomes (personal history of COVID-19, family member diagnosed with or killed by COVID-19, having a partner). Fourth, the use of the DASS-21 could have overestimated the symptoms of depression and anxiety possibly because of the limited instrument specificity. Nevertheless, these results are relevant in order to understand the impact of the COVID-19 pandemic on the mental health of university students and to continue conducting future longitudinal studies.

## 5. Conclusions

We found a high frequency of depression, anxiety, and stress in the university students evaluated. Women who were studying at a public university and who had received training on COVID-19 were associated with a higher prevalence of presenting the three mental health problems evaluated. Meanwhile, students aged 31 and over had a higher prevalence of depression and stress.

## Figures and Tables

**Table 1:** Socio-educational characteristics of participants.

Characteristics	No. (%)
**Age (years)**	
18 to 20	108 (36.9)
21 to 25	136 (46.4)
26 to 30	37 (12.6)
31 and over	12 (4.1)
**Sex**	
Female	226 (77.1)
Male	67 (22.9)
**Civil status**	
Married	13 (4.4)
Cohabitant	13 (4.4)
Single	266 (90.8)
Widower	1 (0.3)
**University type**	
Private	155 (52.9)
Public	138 (47.1)
**Schooling**	
First	102 (34.8)
Second	37 (12.6)
Third	44 (15.0)
Quarter	31 (10.6)
Fifth	24 (8.2)
Sixth	55 (18.8)
**Career**	
Architecture	77 (26.3)
Health Sciences	172 (58.7)
Administration and trade	21 (7.2)
Agricultural	8 (2.7)
Social Sciences	6 (2.1)
Engineering	7 (2.4)
Technology	1 (0.3)
Physical	1 (0.3)
**Training received from COVID-19**	
1	135 (46.1)
2	60 (20.5)
3	31 (10.6)
4	17 (5.8)
5	3 (1.0)
more than 5	47 (16.0)
**Health insurance**	
Social Security (IPS)	19 (6.5)
Private sector	122 (41.6)
Other	152 (51.9)
**People with whom you lived in the home**	
1	15 (5.1)
2	34 (11.6)
3	75 (25.6)
4	59 (20.1)
5	64 (21.8)
more than 5	46 (15.7)
**He was born in the same city as his/her university**	
No	209 (71.3)
Yes	84 (28.7)
**Anxiety**	
Normal	37 (12.6)
Mild	14 (4.8)
Moderate	55 (18.8)
Severe	41 (14.0)
Extreme severe	146 (49.8)
**Depression**	
Normal	74 (25.3)
Mild	27 (9.2)
Moderate	89 (30.4)
Severe	47 (16.0)
Extreme severe	56 (19.1)
**Stress**	
Normal	126 (43.0)
Mild	70 (23.9)
moderate	36 (12.3)
Severe	52 (17.8)
Extreme severe	9 (3.1)

**Table 2:** Factors associated with depression, anxiety and stress.

Variables	*Depression*		*p* *	*Anxiety*		*p* *	*Stress*		*p* *
	No (*n* = 74)	Yes (*n* = 219)		No (*n* = 37)	Yes (*n* = 256)		No (*n* = 126)	Yes (*n* = 167)	
	*n* (%)	*n* (%)		*n* (%)	*n* (%)		*n* (%)	*n* (%)	
**Age (years)**			**0.002**			0.087			**0.030**
18 to 20	15 (13.9)	93 (86.1)		9 (8.3)	99 (91.7)		38 (35.2)	70 (64.8)	
21 to 25	39 (28.7)	97 (71.3)		17 (12.5)	119 (87.5)		63 (46.3)	73 (53.7)	
26 to 30	14 (37.8)	23 (62.2)		9 (24.3)	28 (75.7)		22 (59.5)	15 (40.5)	
31 and over	6 (50.0)	6 (50.0)		2 (16.7)	10 (83.3)		3 (25.0)	9 (75.0)	
Sex			**<0.001**			**<0.001**			**<0.001**
Female	42 (18.6)	184 (81.4)		12 (5.3)	214 (94.7)		79 (35.0)	147 (65.0)	
Male	32 (47.8)	35 (52.2)		25 (37.3)	42 (62.7)		47 (70.2)	20 (29.9)	
Single marital status			**0.016**			0.720			0.874
No	12 (44.4)	15 (55.6)		4 (14.8)	23 (85.2)		12 (44.4)	15 (55.6)	
Yes	62 (23.3)	204 (76.7)		33 (12.4)	233 (87.6)		114 (42.9)	152 (57.1)	
University type			**<0.001**			**0.003**			**0.004**
Private	55 (35.5)	100 (64.5)		28 (18.1)	127 (81.9)		79 (51.0)	76 (49.0)	
Public	19 (13.8)	119 (86.2)		9 (6.5)	129 (93.5)		47 (34.1)	91 (65.9)	
Health career			0.129			0.126			0.334
No	25 (20.7)	96 (79.3)		11 (9.1)	110 (90.9)		48 (39.7)	73 (60.3)	
Yes	49 (28.5)	123 (71.5)		26 (15.1)	146 (84.9)		78 (45.4)	94 (54.7)	
Training received from COVID-19			0.194			0.372			**0.026**
1	26 (19.3)	109 (80.7)		12 (8.9)	123 (91.1)		59 (43.7)	76 (56.3)	
2	21 (35.0)	39 (65.0)		11 (18.3)	49 (81.7)		27 (45.0)	33 (55.0)	
3	9 (29.0)	22 (71.0)		3 (9.7)	28 (90.3)		12 (38.7)	19 (61.3)	
4	4 (23.5)	13 (76.5)		3 (17.7)	14 (82.4)		13 (76.5)	4 (23.5)	
5	0 (0.0)	3 (100.0)		0 (0.0)	3 (100.0)		0 (0.0)	3 (100.0)	
more than 5	14 (29.8)	33 (70.2)		8 (17.0)	39 (83.0)		15 (31.9)	32 (68.1)	
health insurance			0.063			0.250			0.199
Social Security (IPS)	8 (42.1)	11 (57.9)		4 (21.1)	15 (78.9)		11 (57.9)	8 (42.1)	
Other	31 (20.4)	121 (79.6)		15 (9.9)	137 (90.1)		59 (38.8)	93 (61.2)	
Private sector	35 (28.7)	87 (71.3)		18 (14.8)	104 (85.3)		56 (45.9)	66 (54.1)	
People with whom you lived in the home			0.128			0.954			0.669
1	8 (53.3)	7 (46.7)		1 (6.7)	14 (93.3)		5 (33.3)	10 (66.7)	
2	9 (26.5)	25 (73.5)		5 (14.7)	29 (85.3)		16 (47.1)	18 (52.9)	
3	14 (18.7)	61 (81.3)		11 (14.7)	64 (85.3)		31 (41.3)	44 (58.7)	
4	17 (28.8)	42 (71.2)		7 (11.9)	52 (88.1)		29 (49.2)	30 (50.9)	
5	15 (23.4)	49 (76.6)		7 (10.9)	57 (89.1)		29 (45.3)	35 (54.7)	
more than 5	11 (23.9)	35 (76.1)		6 (13.0)	40 (87.0)		16 (34.8)	30 (65.2)	
He was born in the same city as his university			0.718			0.352			0.453
No	54 (25.8)	155 (74.2)		24 (11.5)	185 (88.5)		87 (41.6)	122 (58.4)	
Yes	20 (23.8)	64 (76.2)		13 (15.5)	71 (84.5)		39 (46.4)	45 (53.6)	

* Categorical variables calculated with the chi-square test.

**Table 3:** Factors associated with depression, anxiety, and stress in multiple regression analysis.

Characteristics	*Depression*					*Anxiety*							*Stress*					
	Simple Regression			Multiple Regression		Simple Regression				Multiple Regression			Simple Regression			Multiple Regression		
	PR	CI 95%	*p* *	PR	CI 95%	*p* *	PR	CI 95%	*p* *	PR	CI 95%	*p* *	PR	CI 95%	*p* *	PR	CI 95%	*p* *
**Age (years)**																		
18 to 20	Ref.			Ref.			Ref.			Ref.			Ref.			Ref.		
21 to 25	0.83	0.73–0.93	**0.002**	0.93	0.82–1.05	0.257	0.95	0.88–1.04	0.281	1.03	0.96–1.11	0.386	0.83	0.73–0.94	**0.004**	0.91	0.79–1.04	0.179
26 to 30	0.72	0.60–0.86	**<0.001**	0.82	0.70–0.95	**0.011**	0.83	0.67–1.02	**0.077**	0.92	0.77–1.09	0.312	0.63	0.52–0.76	**<0.001**	0.72	0.51–1.01	0.059
31 and over	0.58	0.45–0.75	**<0.001**	0.70	0.65–0.76	**<0.001**	0.91	0.85–0.97	**0.004**	1.01	0.92–1.12	0.799	1.16	1.06–1.27	**0.002**	1.29	1.15–1.43	**<0.001**
**Sex**																		
Female	Ref.			Ref.			Ref.			Ref.			Ref.			Ref.		
Male	0.64	0.44–0.94	**0.022**	0.67	0.47–0.95	**0.026**	0.66	0.57–0.77	**<0.001**	0.67	0.58–0.76	**<0.001**	0.46	0.37–0.57	**<0.001**	0.47	0.37–0.60	**<0.001**
**Single marital status**																		
No	Ref.			Ref.			Ref.						Ref.					
Yes	1.38	1.07–1.78	**0.012**	1.17	0.86–1.58	0.310	1.03	0.90–1.18	0.689				1.03	0.82–1.29	0.809			
**Health career**																		
No	Ref.						Ref.						Ref.					
Yes	0.90	0.80–1.01	0.073				0.93	0.85–1.02	0.148				0.91	0.82–1.00	0.054			
**University type**																		
private	Ref.			Ref.			Ref.			Ref.			Ref.			Ref.		
public	1.34	1.16–1.54	**<0.001**	1.31	1.09–1.58	**0.003**	1.14	1.12–1.16	**<0.001**	1.16	1.09–1.24	**<0.001**	1.34	1.12–1.61	**0.001**	1.35	1.08–1.68	**0.009**
**Training received from COVID-19**																		
1	Ref.			Ref.			Ref.			Ref.			Ref.			Ref.		
2	0.81	0.63–1.03	0.083	0.90	0.75–1.08	0.241	0.90	0.76–1.06	0.198	0.94	0.83–1.06	0.295	0.98	0.69–1.38	0.894	1.09	0.80–1.49	0.590
3	0.88	0.73–1.06	0.188	1.07	0.90–1.27	0.428	0.99	0.90–1.10	0.866	1.06	0.99–1.13	**0.082**	1.09	0.86–1.38	0.488	1.33	0.91–1.95	0.144
4	0.95	0.75–1.19	0.644	1.24	0.93–1.66	0.136	0.90	0.66–1.23	0.522	1.02	0.80–1.30	0.890	0.42	0.23–0.77	**0.005**	0.57	0.30–1.09	0.089
5	1.24	1.09–1.41	**0.001**	1.56	1.20–2.02	**0.001**	1.10	1.01–1.19	**0.025**	1.12	1.06–1.19	<0.001	1.78	1.41–2.23	**<0.001**	1.74	1.27–2.40	**0.001**
more than 5	0.87	0.70–1.08	0.205	1.06	0.90–1.25	0.498	0.91	0.78–1.06	0.239	0.96	0.90–1.06	0.555	1.21	0.96–1.52	0.106	1.43	1.19–1.73	**<0.001**
**health insurance**																		
Social Security (IPS)	Ref.						Ref.						Ref.			Ref.		
Private sector	1.23	0.69–2.20	0.481				1.14	0.90–1.45	0.277				1.28	0.85–1.94	0.235	1.13	0.73–1.76	0.580
Other	1.38	0.83–2.28	0.217				1.08	0.87–1.34	0.489				1.45	1.02–2.08	**0.041**	1.28	0.88–1.85	0.192
**People with whom you lived in the home**																		
1	Ref.						Ref.			Ref.			Ref.					
2	1.58	0.85–2.91	0.147				0.91	0.70–1.20	0.516	0.92	0.72–1.16	0.464	0.79	0.55–1.16	0.229			
3	1.74	0.85–3.57	0.128				0.91	0.84–0.99	**0.049**	0.91	0.79–1.03	0.144	0.88	0.61–1.26	0.486			
4	1.53	0.71–3.26	0.276				0.94	0.79–1.13	0.527	0.99	0.81–1.22	0.959	0.76	0.48–1.20	0.245			
5	1.64	0.76–3.53	0.205				0.95	0.82–1.11	0.554	0.94	0.80–1.10	0.423	0.82	0.58–1.15	0.252			
more than 5	1.63	0.64–4.12	0.302				0.93	0.75–1.16	0.527	0.91	0.74–1.12	0.366	0.98	0.67–1.43	0.909			
**He was born in the same city as his university**																		
No	Ref.						Ref.						Ref.					
Yes	1.03	0.85–1.24	0.778				0.95	0.88–1.04	0.294				0.92	0.78–1.07	0.287			

* *p*-values obtained with generalized linear models (GLM), Poisson family, log link function, robust variance, and cluster by year of studies.

## Data Availability

The dataset generated and analyzed during the current study is not publicly available because the ethics committee has not provided permission/authorization to publicly share the data but are available from the corresponding author on reasonable request.
